# Multistrategy Improved Sparrow Search Algorithm Optimized Deep Neural Network for Esophageal Cancer

**DOI:** 10.1155/2022/1036913

**Published:** 2022-09-27

**Authors:** Yanfeng Wang, Qing Liu, Junwei Sun, Lidong Wang, Xin Song, Xueke Zhao

**Affiliations:** ^1^School of Electrical and Information Engineering, Zhengzhou University of Light Industry, Zhengzhou, China; ^2^State Key Laboratory of Esophageal Cancer Prevention & Treatment and Henan Key Laboratory for Esophageal Cancer Research of the First Affiliated Hospital, Zhengzhou University, Zhengzhou 450052, China

## Abstract

Deep neural network is a complex pattern recognition network system. It is widely favored by scholars for its strong nonlinear fitting ability. However, training deep neural network models on small datasets typically realizes worse performance than shallow neural network. In this study, a strategy to improve the sparrow search algorithm based on the iterative map, iterative perturbation, and Gaussian mutation is developed. This optimized strategy improved the sparrow search algorithm validated by fourteen benchmark functions, and the algorithm has the best search accuracy and the fastest convergence speed. An algorithm based on the iterative map, iterative perturbation, and Gaussian mutation improved sparrow search algorithm is designed to optimize deep neural networks. The modified sparrow algorithm is exploited to search for the optimal connection weights of deep neural network. This algorithm is implemented for the esophageal cancer dataset along with the other six algorithms. The proposed model is able to achieve 0.92 under all the eight scoring criteria, which is better than the performance of the other six algorithms. Therefore, an optimized deep neural network based on an improved sparrow search algorithm with iterative map, iterative perturbation, and Gaussian mutation is an effective approach to predict the survival rate of esophageal cancer.

## 1. Introduction

Esophageal cancer is a malignant tumor with high incidence, high mortality, and high recurrence rate [1]. The way of cancer patient treatment is mainly based on physicians' experience, which inevitably leads to physicians' treatment errors [2]. How to effectively forecast the survival time of esophageal cancer patients to decrease the misdiagnosis rate of physicians is a current research hotspot [3–6]. In recent years, the quick advancement of artificial intelligence has enabled the construction of intelligent systems, a simple and easy task [7]. Artificial intelligence has been able to simulate human intelligence more accurately to learn and make predictive actions on medical datasets [8, 9]. In particular, deep learning excels at complex machine learning tasks by building multilayer neural networks [10, 11]. Deep learning has made remarkable progress and excellent performance in areas such as medical image process, biological image process, and target inspection completion [12–15]. Since deep learning is adept at handling complex nonlinear problems, it can perform comparably or even better than professional physicians in the fields of disease identification and disease prediction [16, 17]. Therefore, survival prediction models based on deep learning will hopefully provide a scientific basis for clinical medical decisions in esophageal cancer.

Neural network [18] is the basis of deep learning, a mathematical simplification of single-layer perception of human nerve cells. Deep neural network (DNN) has been continuously trained by researchers regarding the exploration of the human nervous system with multiple implicit layers [19, 20]. DNN has been used to identify different ECG abnormalities, and its identification results are better than hospitalists with heart disease and emergency medical medicine [21]. DNN model is effective in identifying tumors or hyperplastic polyps, and the identification time of the model is shorter than endoscopy [22]. 3D-DNN has achieved progress in automated lung cancer diagnosis by computed tomography. The model can identify all suspicious lung pathologies and evaluate the grades of lung malignancies [23]. DNN has exhibited state-of-the-art performance in areas such as medical image recognition [24], cancer diagnosis, pathology examination [25], stock price prediction [26], and daily plant transpiration estimation [27]. However, the DNN model trained on small datasets typically exhibits poorer properties than traditional machine learning approaches, such as shallow neural networks and support vector machines.

Gradient descent is a common optimization approach for neural network learning, which cleverly utilizes gradients to find function minima [28]. Nevertheless, when gradient descent is used as an optimization method for DNN, it is usually difficult to get rid of the local minimum value and slow convergence speed. The metaheuristic algorithm can effectively eliminate the above problem by obtaining the optimal solution through global search [29]. Metaheuristic algorithm is extensively implemented in function optimization, fuzzy logic system, and image treatment [30–32]. The DNN model optimized by the Gray Wolf Optimization (GWO) algorithm has been applied to extract and classify features from the CAD image dataset of diabetic retinopathy [33]. The model has advantages in terms of accuracy, precision, recall, sensitivity, and specificity. Gravitational search algorithm (GSA) plays an essential role in enhancing the prediction accuracy of DNN models [34]. The model can more precisely differentiate between benign and malignant nodules in CT scan lung images. Whale Optimization Algorithm (WOA) of DNN has achieved significant results [35]. Dimensionality reduction by principal component analysis (PCA) and firefly algorithm is performed on the diabetic retinopathy dataset [36]. The simplified dataset is sent to the DNN model for classification. The DNN model is shown to outperform other machine learning algorithms in accuracy, precision, recall, sensitivity, and specificity. Optimization algorithm for feature dimensionality reduction is a common neural network optimization method. However, the impact of dimensionality reduction on data results is significant, which may result in DNN performing even worse than traditional methods on small sample datasets [37]. This makes the application of DNN to small sample datasets a challenge. In this study, a method based on a metaheuristic algorithm for reducing the mean square deviation difference of DNN models and changing the interlayer connection weights makes the accuracy of models for small datasets improved.

In this study, the chaotic map is introduced into the sparrow search algorithm (SSA), which allows the sparrow population to be increased in sample diversification and improved in even distribution. The ability to escape local optimum and increase convergence speed is obtained by the SSA. Gaussian mutation and chaotic perturbations are also introduced into the SSA, which makes it possible to adjust the aggregated sparrow individuals. The local search ability of SSA in the focal search region is enhanced by Gaussian mutation and chaotic perturbation. The iterative map is employed in the SSA to verify its optimal performance by fourteen benchmark functions. Iterative map, iterative perturbation, and Gaussian mutation optimized SSA (IIGSSA) are proposed. IIGSSA is adopted to find the connection weights of DNN. To evaluate the algorithm performance, IIGSSA-DNN is applied to the esophageal cancer dataset along with eight predictive classification algorithms, which are DNN, PSO-DNN, GSA-DNN, GWO-DNN, WOA-DNN, SSA-DNN, IIGSSA-K Nearest Neighbor (KNN), and IIGSSA-Support Vector Machine (SVM). They also use 26 metrics as input and survival time as output. Due to the small samples used in this paper, five-fold cross-validation is selected to evaluate the authenticity of the model performance. Ten scoring criteria are used as judging criteria, which are accuracy (Acc), false positive rate (FPR), recall rate (REC), true positive rate (TPR), precision (PRE), true negative rate (TNR), area under the curve (AUC), F1-measure (F1-M), and pooled mean (G-M) [38]. The IIGSSA-DNN model has an FPR value of 0.1, a *P*-value of 0.01, and all other scoring criteria have a value of 0.92. The model is evidenced to possess superior predictive accuracy and statistical value. Therefore, IIGSSA-DNN is an algorithm to accurately predict the survival time for esophageal cancer patients. IIGSSA-DNN is expected as a novel approach for the future clinical treatment of esophageal cancer.

A mixed metaheuristic algorithm is formulated to improve the prediction algorithm of the DNN network structure. The iterative map, iterative perturbation, and Gaussian mutation optimized SSA (IIGSSA) is developed. The optimum network structure of DNN can be defined by IIGSSA. IIGSSA-DNN is expected to be an enabling instrument for the clinical diagnosis and treatment of esophageal cancer. This is also an innovation of IIGSSA-DNN applied to nonimage datasets of esophageal cancer. The primary contributions of this study can be summarized as follows:

1. The iterative map, iterative perturbation, and Gaussian mutation optimized SSA with the fastest convergence rate and best accuracy are validated in fourteen benchmark functions.
2. IIGSSA is used as an optimization strategy to improve the model accuracy by optimizing the optimal connection weights for the DNN model.
3. IIGSSA-DNN is presented to the esophageal cancer dataset, and IIGSSA can predict the survival time of esophageal cancer patients more precisely than other algorithms.

The remainder of this study is structured as follows.  Section 2 introduces the SSA and improvement ideas and identifies the iterative map, iterative perturbation, and Gaussian variation as the best optimization strategy on fourteen benchmark functions. Section 3 describes the principles and improvement ideas of DNN and applies IIGSSA-DNN to the esophageal cancer dataset. The conclusions are described in Section 4.

## 2. Development and Validation of an Improved SSA Based on Iterative Map, Iterative Perturbation, and Gaussian Mutation

### 2.1. SSA

SSA [39, 40] is a swarm intelligence optimization algorithm that simulates sparrow foraging behavior and antipredation behavior. The sparrow population achieves foraging behavior through three task divisions, which are discovery, follow, and alert. Discoverers are leaders in the population due to their high fitness. This is associated with their capacity to seek and provide the location and direction of food resources. Epigones follow and forage around the discoverers for greater fitness. Epigones in a population supervise the behavior of other individuals, and they compete with high-intake peers for food resources to improve their predation rates. When the whole population is threatened by predators or perceives danger, the sparrows immediately counter-hunt. The sparrows in the outer circle of the population are vulnerable to predators, and they need to constantly relocate to the center of the population. The sparrow in the center of the population adjusts its position to keep its distance from others as short as possible. SSA simulates the foraging process of sparrows to obtain the solution for the optimization problem.

Suppose a population of *N* sparrows searches in *D* dimensional search space; then, the position of the *i*_*th*_ sparrow in *D* dimensional search space is X_*i*_=[*x*_*i*1_, *x*_*i*2_, ⋯, *x*_*id*_, ⋯, *x*_*iD*_], *i*=1, 2, ⋯, *N*. *x*_*id*_ stands for the position of the *i*_*th*_ sparrow in $D$ dimension. SSA takes 10%∼20% of the finders in the sparrow population as constraint conditions, and the position update equation is as follows:

(1) $$\begin{array}{c} x_{id}^{t+1}=\left\{\begin{array}{c} x_{id}^{t}\cdot \exp\left(\frac{-i}{\alpha T}\right),{\text{R}}_{2\;}<S_{T},\\ x_{id}^{t}+QL,{\text{R}}_{2}\geq S_{T}, \end{array}\right. \end{array}$$

where *t* is the current iteration number. *T* is the maximum number of iterations. *α* is the uniform random number between (0,  1]. *Q* is a random number that follows the standard normal distribution. *L* is a 1 × *D* dimensional matrix composed of element 1. R_2_ ∈ [0,  1] is the warning value. *S*_*T*_ ∈ [0.5,  1] is the safety value. If R_2_ < *S*_*T*_, predators or other hazards are not near the population, and the current search environment is safe. Discoverers conduct extensive searches to guide the population to obtain higher fitness. If R_2_ ≥ *S*_*T*_, epigones are keen to spot predators and quickly release danger signals for reminding the population to act immediately against predation. The population adjusts its search strategy and quickly moves towards the safe area.

The update equation for follower position is as follows:

(2) $$\begin{array}{c} x_{id}^{t+1}=\left\{\begin{array}{c} Q\cdot \exp\left(\frac{xw_{d}^{t}-x_{id}^{t}}{i^{2}}\right),\;i\;>\frac{n}{2},\\ xb_{d}^{t+1}+\left|x_{id}^{t}-xb_{d}^{t+1}\right|A^{+}\cdot L,\;i\;\leq \frac{n}{2}, \end{array}\right. \end{array}$$

where *A*^+^ is 1 × *D* vector allocation with the value 1 or -1, and*A*^+^=*A*^T^(*AA*^T^)^−1^. *xw*_*d*_^*t*^ is the worst position of sparrows in the *D*dimension in the *t* iteration. *xb*_*d*_^*t*+1^ is the optimal position of sparrows in the *D*dimension at the *t*+1 iteration. If *i*  > *n*/2, the *i*_*th*_ epigone is starving without food. To obtain higher fitness, the epigone shifts to the area for food. If *i*  ≤ *n*/2, the *i*_*th*_ epigone has a better fitness at the current best location, and the epigone randomly seeks a location near the best location for foraging. 10%∼20% of the population is responsible for reconnaissance, and their positions are updated as follows:

(3) $$\begin{array}{c} x_{id}^{t+1}=\left\{\begin{array}{c} xb_{d}^{t+1}+\beta \left(x_{id}^{t}-xb_{d}^{t+1}\right),f_{i}\neq f_{g},\\ x_{id}^{t}+\text{K}\left(\frac{x_{id}^{t}-xw_{d}^{t}}{\left|f_{i}-f_{w}\right|+e}\right),f_{i}=f_{g}, \end{array}\right. \end{array}$$

where *β* is a step size control parameter with the mean value of 0 and variance of 1, which satisfies normal distribution random number. K is a random step size control parameter in the interval [−1,  1], referring to the orientation of the sparrow's flight. *e* is a minimum constant to prevent the numerator being zero. *f*_*i*_ is the fitness value of the *i*_*th*_ sparrow. *f*_*g*_ is the optimal fitness value of the current sparrow population. *f*_*w*_ is the worst fitness value of the current sparrow species. If *f*_*i*_ ≠ *f*_*g*_, sparrows on the edge of the population are vulnerable to predator attack, and they need to position themselves to avoid the attack. If *f*_*i*_=*f*_*g*_, the sparrow is in the middle of the population. To avoid being attacked by predators, sparrows in this area adjust their search strategy by approaching other sparrows in time after realizing the threat of predators.

### 2.2. Chaotic Map, Chaotic Perturbation, and Gaussian Mutation Strategies

Chaotic [41] is a complex nonlinear motion type ubiquitous in nature. This nonlinear phenomenon usually occurs under certain conditions, which makes the ordered trajectory deviates from the original path suddenly into a disorderly form. The chaotic map is favored by scholars because of its randomness, ergodicity, and regularity. This is associated with its capacity to sustain a rich population variety. The chaotic map optimization metaheuristic algorithm enables the algorithm to escape local optimum while gaining higher global search capability. Nine common chaotic maps are listed in this study to optimize SSA.

(1) Tent map [42]:
  (4) $$\begin{array}{c} Z_{k+1}=\left\{\begin{array}{c} Z_{k}/\beta ,Z_{k}\in \left(0,\beta \right],\\ \left(1-Z_{k}\right)/\left(1-\beta \right),Z_{k}\in \left(\beta ,1\right], \end{array}\right. \end{array}$$
where *β* indicates the mapping parameter for the tent. If *β*=0.7, the obtained sequence distribution is more uniform.
(2) Chebyshev map [43]:
  (5) $$\begin{array}{c} Z_{k+1}=\cos\left(\phi {\text{cos}}^{-1}Z_{k}\right), \end{array}$$
where *ϕ* is the order of the Chebyshev map. When *ϕ* is bigger than 2, the Lyapunov exponent is nonnegative and the system is chaotic.
(3) Circle map [44]:
  (6) $$\begin{array}{c} Z_{k+1}=\text{mod}\left(Z_{k}+0.2-\frac{0.5}{2\pi }\sin\left(2\pi Z_{k}\right),1\right), \end{array}$$
where *a*=0.5*b*=2.2.
(4) Iterative map [45]:
  (7) $$\begin{array}{c} Z_{k+1}=\sin\left(\frac{a\pi }{Z_{k}}\right), \end{array}$$
where *a* ∈ (0, 1) is the iterative map control parameter.
(5) Sine map [46]:
  (8) $$\begin{array}{c} Z_{k+1}=\frac{4}{a}\sin\left(\pi Z_{k}\right), \end{array}$$
where the sine map is a single-peak map, whose range is [−1, 1], and the sine map parameter is *a* ∈ (0, 4].
(6) Singer map [47]:
  (9) $$\begin{array}{c} Z_{k+1}=\mu \left(7.86Z_{k}-23.31Z_{k}^{2}+28.75Z_{k}^{3}-13.302875Z_{k}^{4}\right), \end{array}$$
where *μ* is the singer parameter whose value range is (0.9,  1.08). When *Z*_*k*_ ∈ [0, 1], the singer map is distributed in the range [0, 1], which can make a more uniform distribution of *Z*_*k*_.
(7) Sinusoidal map [48]:
  (10) $$\begin{array}{c} Z_{k+1}=aZ_{k}^{2}\sin\left(\pi Z_{k}\right), \end{array}$$
where *a*=2.3, *Z*_0_=0.7.
(8) Logistic map [49]:
  (11) $$\begin{array}{c} Z_{k+1}=\mu Z_{k}\left(1-Z_{k}\right), \end{array}$$
where *μ* is a logistic parameter whose value range is [0, 4]. When *Z*_*k*_ ∈ [0, 1], the logistic map operates in chaos. The nearer the *μ* value gets to 4, the more uniformly *Z*_*k*_ is dispersed between 0 and 1.
(9) Cubic map [50]:
  (12) $$\begin{array}{c} Z_{k+1}=\rho Z_{k}\left(1-Z_{k}^{2}\right), \end{array}$$
where *ρ* ∈ (0, 1) is the Cubic map control parameter. Cubic map is one of the most common chaotic maps.

Chaotic perturbation is the introduction of a random perturbation quantity obeying chaotic distribution based on the original solution. A chaotic variant is derived from the chaotic map, and the chaotic variant is brought into the solution space by (13).

(13) $$\begin{array}{c} X_{n}^{d}=m_{\text{min}}+C\left(m_{\text{max}}-m_{\text{min}}\right)\;, \end{array}$$

where *m*_min_ is the minimum value of the variable *X*_*n*_^*d*^ in d_*th*_ dimension. *m*_max_ is the maximum value of the variable *X*_*n*_^*d*^ in d_*th*_ dimension. *X*_*n*_^*d*^ is the amount of chaotic perturbation generated by the solution in the *m* dimension. *C* is chaotic variable. The chaotic perturbation equation is

(14) $$\begin{array}{c} X_{n}^{\prime }=\frac{\left(X^{\prime }+X_{n}\right)}{2}, \end{array}$$

where *X*′ is the individual requiring chaotic perturbation. *X*_*n*_ is the amount of chaotic perturbation generated. *X*_*n*_′ is the individual after chaotic perturbation.

Gaussian mutation [51] is an optimized strategy modified from a genetic algorithm mutation operation. Gaussian mutation operation generates random numbers obeying normal distribution to generate new positions by acting on the original position vector. It implements neighborhood search instruction in a small range to distribute most of the variational operators in the original position. The advantages of high optimization accuracy and the difficulty of falling into local optimization are gained by the optimization algorithm. A small fraction of mutation operators away from the current position makes the potential region search more advantageous and the population diversity richer. Therefore, Gaussian mutation is exploited to modify the algorithm, which will result in a much faster search speed and a much faster convergence trend. The Gaussian probability density equation is as follows:

(15) $$\begin{array}{c} \text{M}\left(x\right)=x\left(1+\text{G}\left(0,1\right)\right), \end{array}$$

where *x* is the initial parameter value and G is the Gaussian normally displaced stochastic number with an expectation value of 0 and a standard deviation of 1.

### 2.3. An Improved SSA

SSA is an algorithm with simple structure, easy implementation, few control parameters, and strong local searchability. It obtains the initial position of the sparrow based on the random initialization method. Although this approach ensures the randomness of the initial positions, the optimal values of the initial positions of some individuals are too different from the actual optimal values, which reduces the convergence speed and the accuracy of the solution. The blind production of initial positions is prone to the phenomenon of overlapping aggregation of initial solutions. It will lead to a low probability of solution space coverage and a low rate of change of population individuals. The pseudorandom number generator is an ideal information source with excellent statistics and stochastic properties. The chaotic map has high randomness and easy implementation, and it can randomly generate chaotic numbers between 0 and 1. Therefore, chaotic maps are ideal for pseudorandom number generators. The introduction of chaotic map in SSA can effectively improve the initialization population blindness problem of the algorithm. The introduction of the chaotic map can effectively increase the global search capability of SSA. Gaussian mutation optimization strategy can strengthen the local search capability of the population and improve search accuracy. To protect against the solution stagnation phenomenon caused by the emergence of local optimum, the chaotic perturbation strategy is introduced into SSA. Some local optimal individuals are endowed by chaotic perturbations with a “new dynamism” capable of stepping outside the local optimum. The optimization strategy in SSA directly affects the convergence precision, search capability, and velocity. Strategy selection is crucial to the performance of SSA. In this study, a chaotic Gaussian sparrow search algorithm (CGSSA) based on a multistrategy fusion mechanism is developed by introducing a chaotic map, Gaussian mutation, and chaotic perturbation strategies. The detailed steps of the CGSSA execution are described below.

**Step 1 .**

Initialize the population size *N*, the number of discoverers *P*_*a*_, the number of scouting warning sparrows *S*_*a*_, the dimensionality of the objective function *D*, the upper bound *ub*, lower bound *lb* of the initial value, and the maximum number of iterations *T*.

**Step 2 .**

Initialize the sparrow population by chaotic sequences to generate *N∗D* dimensional vectors *Z*. Each component of *Z* is brought into a defined range of values by equations (4)–(12).

**Step 3 .**

Calculate the fitness *f*_*i*_ of each sparrow, select the current optimal fitness *f*_*b*_ and its correspondent position *X*_*b*_ of each sparrow.

**Step 4 .**

Select the top *P*_*a*_ sparrows with the best fitness as discoverers and the rest as followers. Update the positions of discoverer and follower according to (1) and (2).

**Step 5 .**

Randomly select *S*_*a*_ sparrows from the sparrow population as reconnaissance alerts. Update their positions according to (3).

**Step 6 .**

Recompute the fitness value of individual sparrows and the average fitness value of the sparrow population after each iteration.

**Step 7 .**

If *f*_*i*_ < *f*_*a*_, perform a Gaussian mutation operation on the aggregated sparrow population according to (15). Compare the postmutation individuals with the premutation individuals. Determine whether to accept the position of the postmutation sparrow individual. If *f*_*i*_ ≥ *f*_*a*_, perform a chaotic perturbation operation on the dispersed population of sparrows by (4)–(12). Compare postdisturbance individuals with predisturbance individuals. Determine whether to accept the location of the postperturbation individual sparrows.

**Step 8 .**

Get the current state of the sparrow population. Update the optimal position *X*_*b*_ and its fitness *f*_*b*_ by the whole sparrow population.

**Step 9 .**

If the algorithm runs to the maximum number of iterations, end the loop and output the search results. Otherwise, return to Step 4.

Chaotic map strategy is used by CGSSA to initialize the population to improve the population diversity. Both Gaussian mutation and chaotic perturbation strategies are introduced into the SSA, targeting to solve the sparrow divergence and aggregation problems. The local search ability of SSA in the focal search region is enhanced by Gaussian mutation and chaotic perturbation. To find the optimal chaotic map and chaotic perturbation strategies, this study tests the performance of nine chaotic map and chaotic perturbation strategies combined with the Gaussian mutation strategy to optimize SSA, respectively. The benchmark functions are chosen for the test functions, respectively.

### 2.4. Benchmark Functions Test

**Figure 1:**
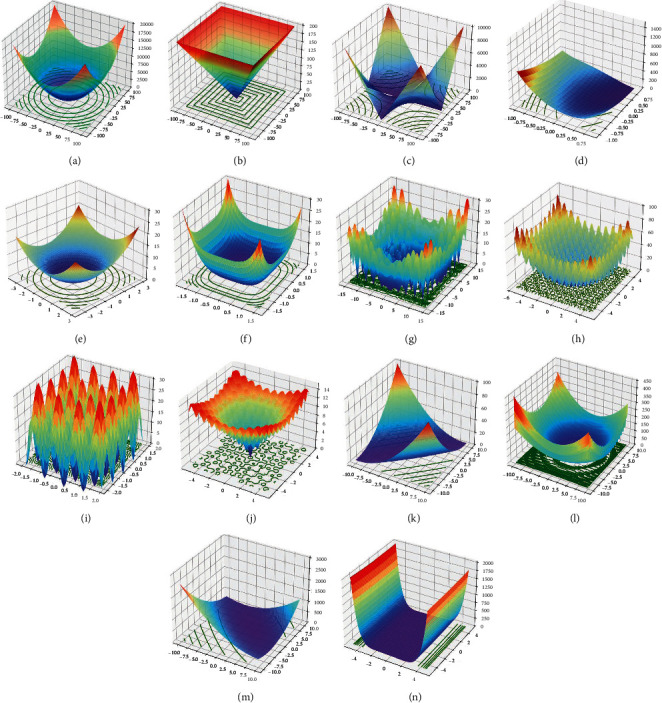
Solution space diagram for nineteen benchmark functions. (a) Sphere function. (b) Schwefel 2.21 function. (c) Schwefel 2.22 function. (d) Rosenbrock function. (e) Step function. (f) Quartic function. (g) Alpine function. (h) Rastrigin function. (i) Sum squares function. (j) Ackley function. (k) Maytas function. (l) Levi function. (m) Booth function. (n) Three-Hump function.

To select the chaotic map with the highest adaptation to the SSA, fourteen benchmark functions [52–54] are selected in this study. The original SSA and the nine improved algorithms are validated. The fourteen benchmark functions are given in Table 1. The solution space diagram of the fourteen benchmark functions is illustrated in Figure 1. The parameters of the 10 algorithms are set as follows.

**Figure 2:**
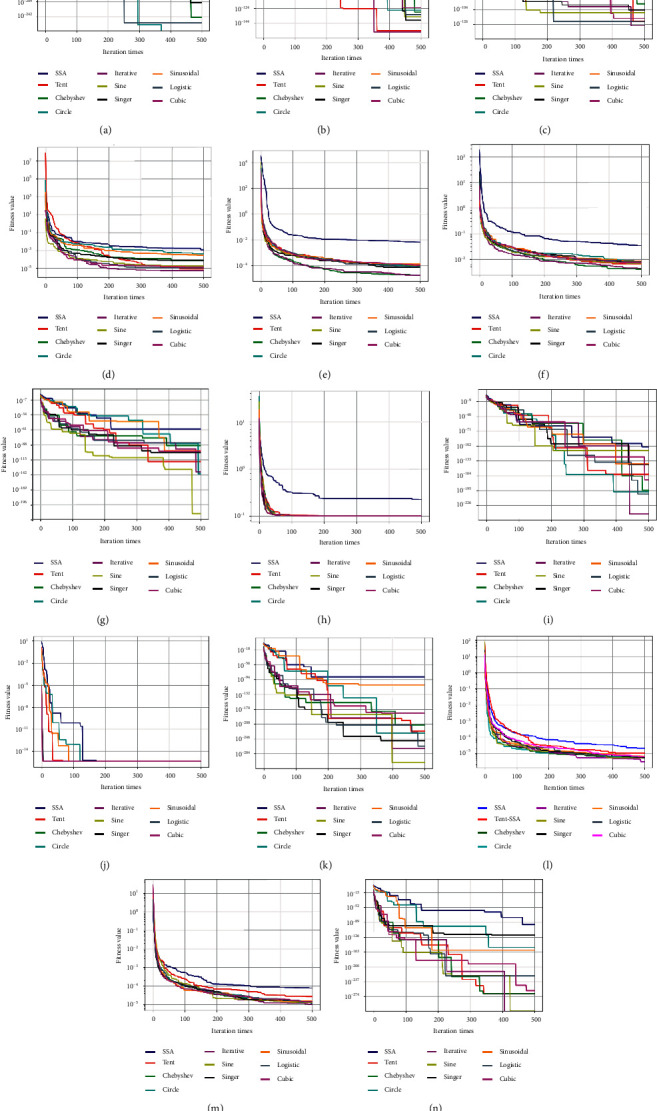
Comparison of convergence curves of 10 algorithms on benchmark functions. (a) Sphere function. (b) Schwefel 2.21 function. (c) Schwefel 2.22 function. (d) Rosenbrock function. (e) Step function. (f) Quartic function. (g) Alpine function. (h) Rastrigin function. (i) Sum squares function. (j) Ackley function. (k) Maytas function. (l) Levi function. (m) Booth function. (n) Three-Hump function.

The number of populations is 30. The maximum number of iterations is 500. The dimension of the objective function and the range of initial values are kept consistent with Table 1. The number of discoverers and followers is set to 20% of the sparrow population size. To avoid contingency in the search outcomes, each benchmark function is examined 20 times individually. The optimal value, mean, and standard deviation of the run results are assessed to determine the robustness of each algorithm. Test outcomes of fourteen benchmark functions are listed in Table 2. The bolded data in the table indicate the best value of each function. A comparison of convergence curves of 10 algorithms on benchmark functions is illustrated in Figure 2.

From Table 2 and Figure 2, no matter which chaotic strategy is chosen to improve SSA, its convergence accuracy is better than the traditional SSA. CGSSA is proved to be effective and feasible. Seven functions obtained theoretical optimal values in ten algorithm tests, which are *F*_1_, *F*_2_, *F*_3_, *F*_7_, *F*_9_, *F*_11_, and *F*_14_. Ten algorithms fall into the same local optimum when testing functions *F*_8_ and *F*_10_. In addition to this, the Chebyshev map achieves a minimum optimum on function *F*_6_. The iterative map obtains a minimum optimum on functions *F*_4_ and *F*_5_. The sine map reaches a minimum optimum on two functions, which are *F*_12_ and *F*_13_. The iterative map and sine map strategies have the highest number of minimum optimums, which means that these two strategies perform best in terms of optimal solutions.

The mean and standard deviation are a pair of statistical indicators describing the overall characteristics of the data. The mean reaction dataset trend is the standard deviation reaction dataset trend. The overall characteristics of the data can be obtained more comprehensively and accurately by means and standard deviations. From the mean and standard deviation analysis, ten algorithms acquire the smallest mean and standard deviation when testing function *F*_10_. Except for the sinusoidal map, the remaining nine algorithms obtain the smallest standard deviation on function *F*_11_. Seven algorithms obtained the smallest standard deviation on *F*_1_, which are SSA, Chebyshev map, circle map, iterative map, sine map, singer map, and logistic map. Five algorithms obtained the smallest standard deviation on function *F*_11_. Seven algorithms obtained the smallest standard deviation on *F*_1_, which are SSA, Chebyshev map, circle map, iterative map, sine map, singer map, and logistic map. Five algorithms obtained the smallest standard deviation on *F*_9_, which are Chebyshev map, circle map, iterative map, logistic map, and cubic map. Six algorithms obtained the smallest standard deviation on *F*_14_, which are SSA, Chebyshev map, iterative map, sine map, logistic map, and cubic map. In addition to this, the Chebyshev map reaches a smallest standard deviation on *F*_8_. Sine map acquires the smallest standard deviation on two functions, which are *F*_7_ and *F*_11_. Iterative map acquires the smallest standard deviation on seven functions, which are *F*_2_, *F*_3_, *F*_4_, *F*_5_, *F*_6_, *F*_12_, and *F*_13_. Eight algorithms fall into the same mean when testing functions *F*_8_, which are Chebyshev map, circle map, iterative map, sine map, singer map, sinusoidal map, logistic map, and cubic map. In addition to this, the Chebyshev map reaches a smallest mean on *F*_11_. Circle map acquires the smallest mean on *F*_1_. Sine map acquires the smallest mean on *F*_7_. Iterative map acquires the smallest standard deviation on nine functions, which are *F*_2_, *F*_3_, *F*_4_, *F*_5_, *F*_6_, *F*_9_, *F*_12_, *F*_13_, and *F*_14_. The minimum means and minimum standard deviations of iterative map strategy are the highest. Iterative map strategy is best played in the standard deviation of the average value. Therefore, iterative map, iterative perturbation, and Gaussian mutation optimized SSA (IIGSSA) are identified for subsequent studies.

## 3. Development and Evaluation of IGSSA-DNN Model for Esophageal Cancer Dataset

### 3.1. Deep Neural Network

DNN is a method for learning the neural structure of the brain to mimic the processing of information [55, 56]. The DNN structure is comprised by multiple perceptrons, also known as multilayer feedforward neural network. DNN has strong learning capability, self-learning capability, and self-adaptive capability. DNN is a complex pattern recognition network system capable of simulating more complex models or representing more abstract relationships of things. DNN is appropriate for big data analysis, which is reflected in disease prediction, medical image recognition, cancer diagnosis, etc.

**Figure 3:**
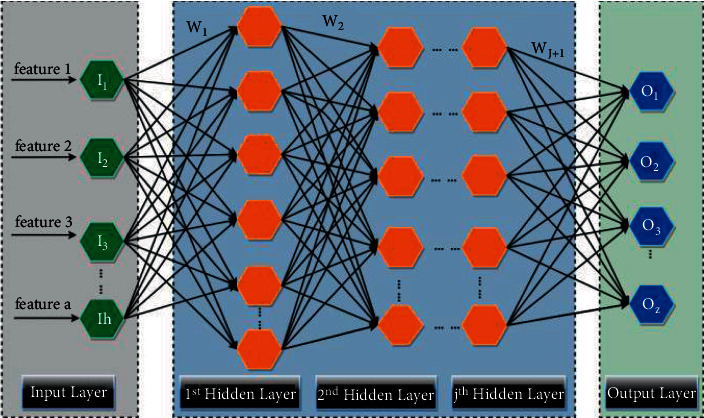
DNN architecture.

From the structural observation, DNN can be divided into an input layer, a multilayer hidden layers, and an output layer, as illustrated in Figure 3. I_1_, I_2_, ..., I_h_ stands the input layer, which is intended to accept messages from exterior devices or systems. The j layer hidden layer effectively assumes the task of the computational engine in the entire network. O_1_, O_2_, ..., O_z_ stands for the output layer, which enables to take decisions on the inputs. h indicates the amount of the input layer's neurons. z means the amount of the output layer's neurons. The output of each vector in the network layer is expressed by (16).

(16) $$\begin{array}{c} {\text{Y}}_{m,n}=f\left({\text{W}}_{m,n}^{\text{T}}{\text{X}}_{m-1}+BH_{m-1}\right), \end{array}$$

where Y_*m*,*n*_ is the output value of the *n* biased neurons at layer *m*, and W_*m*,*n*_^T^ is the weight value of the *n* biased neurons at layer *m*. X_*m*−1_ is the output value of all neurons of *m* − 1 layer, B is the biased neuron of *m* − 1 layer, and *f*(•) is the activation function.

To enhance the representativeness and versatility of the model, activation functions are introduced into DNN to perform nonlinear transformations on the inputs. In this study, the Sigmoid function as the activation function of the DNN is elected to prevent data scattering during transmission [57]. *x* is set as the input. The Sigmoid function is expressed in (17).

(17) $$\begin{array}{c} Sigmoid\left(x\right)=\frac{1}{1+e^{x}}. \end{array}$$

The training procedure of DNN follows the five steps:

1. Define the network structure including the features of the input and output layers, the number of nodes in the network layer, and the implicit layer.
2. Randomly generate initialized weights and biases.
3. Forward propagation to obtain the predicted values.
4. Calculate the loss function and prediction error for forwarding propagation.
5. Reverse propagation to update the weights and biases.

Until the loss function is minimized and the training data is not trained by overfitting, the optimal model is obtained. Therefore, weights play a crucial role in DNN training. The loss function is a fundamental criterion to judge the performance of a neural network. The loss function is sensitive to small changes in weights and biases. The susceptibility of the loss function renders it the best way to find the best weight parameter for DNN [58]. The interlayer weights of the DNN model are optimized by adjusting the error rate, and the adaptability of the model to the current dataset is adjusted, which has a substantial gain on the accuracy of the DNN model output. Mean square error, cross-entropy error, and root mean square error are common loss functions. In this study, the mean square error (MSE) is employed to calculate the error rate, as shown in

(18) $$\begin{array}{c} MSE=\frac{1}{m}\overset{m}{\underset{k=1}{\sum }}{\left(O_{k}-A_{k}\right)}^{2}, \end{array}$$

where *m* is the sample number in the training dataset. *o*_*k*_ is the model output produced by the *k*^*th*^ input. *A*_*k*_ is the *K*^*th*^ actual output. The DNN model is trained by data inputs, and the weight values are obtained to predict the output results.

## 4. An Improved DNN Based on IGSSA

**Figure 4:**
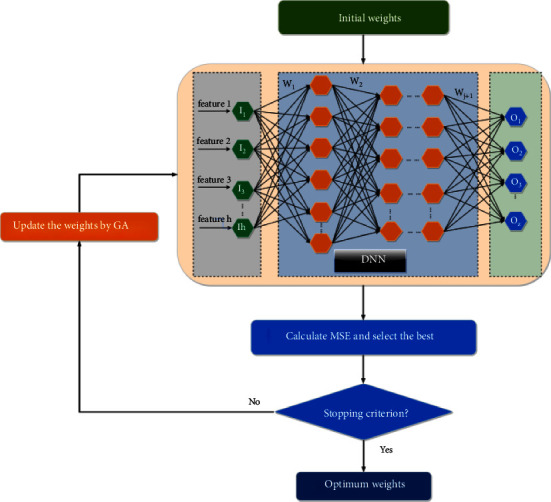
Training phase of the proposed IGSSA for DNN.

The optimal weights of a DNN have a direct correlation with the prediction results of the model. In this study, the accuracy of IGSSA has been verified in Section II, and thus a method to improve DNN based on IIGSSA is proposed. DNN mixed with the proposed IIGSSA is applied to determine the optimal weights of DNN, which will help to enhance the efficiency of the model. The optimum solution of all sparrows in IIGSSA algorithm is exploited to renew the location, and the optimum solution can be reached by the highest number of iterations. The initial weight values are stochastically produced depending on the specified range. The amount of weights of the DNN stands for the number of dimensions of IIGSSA. MSE is chosen as the fitness function of IIGSSA. The training phase of the proposed IGSSA for DNN is displayed in Figure 4. The AUC values are used to test the performance of the evaluated DNN.

### 4.1. Dataset Analysis

The clinical dataset and biological sample dataset of esophageal cancer patients are selected for this study. The dataset is obtained from the State Key Laboratory of Esophageal Cancer Control in Henan Province. A total of 398 patients in the available dataset have complete information, and they are all diagnosed with esophageal cancer in 2007. Continuous metrics in the dataset are shown in Table 3. Discrete metrics in the dataset are indicated in Table 4.

### 4.2. Performance Evaluation

**Figure 5:**
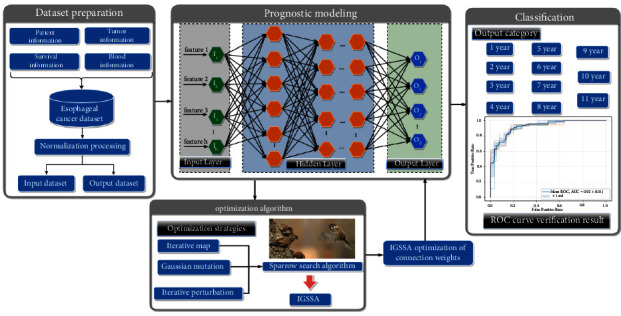
The overall flow chart of IGSSA-DNN.

In this study, a modeling method of optimizing DNN through IIGSSA is proposed. Seventeen blood indicators are provided in the existing esophageal cancer dataset, namely WBC, LY, MONO, NEUT, EOS, BASO, RBC, HB, PLT, TP, ALB, GLB, PT, APTT, TT, and FIB. Seven items of tumor information are supplied, namely tumor length, tumor width, tumor thickness, degree of differentiation, tumor location, transfer situation, and TNM stages. The two physical characteristics are age and gender. Use these 26 features as the input dataset, and the IIGSSA-DNN algorithm is used to build a prognostic model. The overall flow chart of IIGSSA-DNN is illustrated in Figure 5. The robustness and accuracy of IIGSSA-DNN are validated by this study. The IIGSSA-DNN is evaluated against existing scoring criteria to measure the strengths and weaknesses of the proposed algorithm. The most commonly available rubrics [59] are Acc, FPR, REC, TPR, PRE, TNR, AUC, F1-M, and G-M. To avoid the limitations and specificity of fixed division datasets, five-fold cross-validation is employed for objective evaluation of the model.

**Figure 6:**
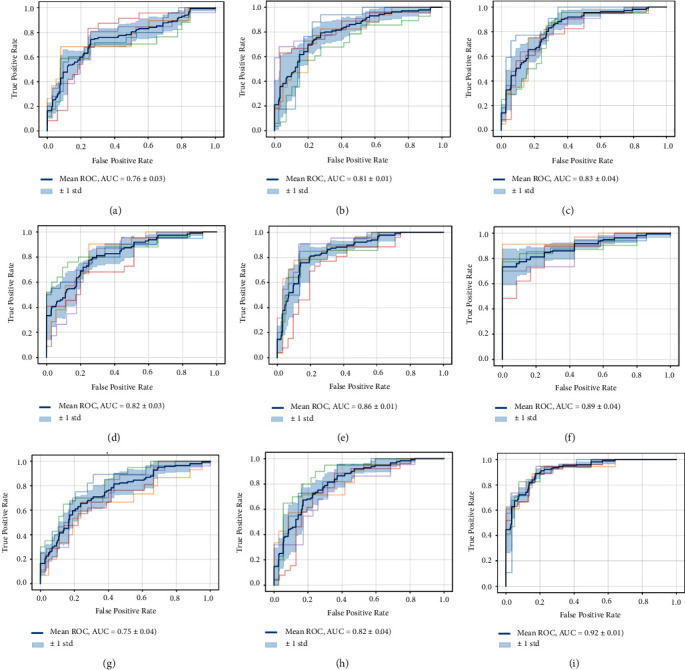
Five ROC curves of nine models. (a) Five ROC curves of DNN. (b) Five ROC curves of PSO-DNN. (c) Five ROC curves of GSA-DNN. (d) Five ROC curves of GWO-DNN. (e) Five ROC curves of WOA-DNN. (f) Five ROC curves of SSA-DNN. (g) Five ROC curves of IGSSA-KNN. (h) Five ROC curves of IGSSA-SVM. (i) Five ROC curves of IGSSA-DNN.

**Figure 7:**
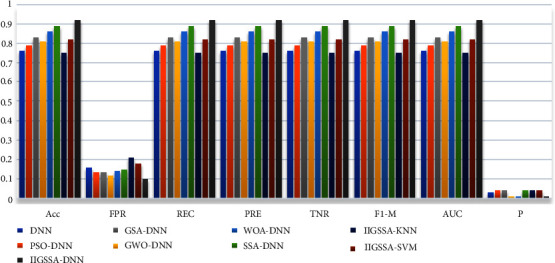
Test accuracy of nine models.

The survival time of esophageal cancer patients is predicted by the proposed IIGSSA-DNN. Five optimization algorithms have been achieved well in the field of neural networks [60–63], which are particle swarm algorithm (PSO), GWO, GSA, WOA, and SSA. Five optimization algorithms are also used to optimize the DNN and tested on the esophageal cancer dataset in comparison with IIGSSA-DNN. To further verify the classification performance of IIGSSA-DNN, SVM and KNN are used to compare with IIGSSA-DNN. KNN and SVM are two machine learning algorithms widely used in the diagnosis of breast cancer [64]. Python is the operating platform of algorithms mentioned in this paper. The deep learning framework PyTorch is employed to implement DNN partial training. 26 features are set as the input dataset, survival time, and survival state as the output dataset, and a DNN model with 10 layers and 128 nodes is constructed. The Sigmoid function is adopted as the activation function of the DNN. The learning rate of the model is fixed to 0.001 [65]. The learning rate is adaptively and randomly adjusted by Adam algorithm. The first-order moment estimates and second-order moment estimates of the gradient are calculated by Adam algorithm [66] to adjust the learning rates of the different parameters. In each iteration, the learning rate is limited to a rough range, which makes the parameters more stable. IIGSSA is employed to enable minimizing the MSE to find the optimal weight value. The population size is set to 30, and the maximum number of iterations is set to 1000. The results of the predictive model assessment are shown in Table 5. The five ROC curves of nine models are displayed in Figure 6. Five ROC curves of DNN are expressed in Figure 6(a). Five ROC curves of PSO-DNN are indicated in Figure 6(b). Five ROC curves of GSA-DNN are illustrated in Figure 6(c). Five ROC curves of GWO-DNN are depicted in Figure 6(d). Five ROC curves of WOA-DNN are demonstrated in Figure 6(e). Five ROC curves of SSA-DNN are seen in Figure 6(f). Five ROC curves of IIGSSA-KNN are expressed in Figure 6(g). Five ROC curves of IIGSSA-SVM are indicated in Figure 6(h). Five ROC curves of IIGSSA-DNN are represented in Figure 6(i). Test accuracy of nine models is shown in Figure 7.

From Table 5, the performance of IIGSSA-DNN for training esophageal cancer dataset is proved to be better than other optimization algorithms. The accuracy of the proposed IIGSSA-DNN is 0.92. The accuracy of IIGSSA-DNN is outperformed by other optimization algorithms of hybrid DNN. The patient's survival time is well predicted by IIGSSA-DNN. REC is the probability of correctly classifying positive samples. PRE is the proportion of samples with positive classification results versus those which are actually positive. REC and PRE are a good way to determine the number of accurate samples for classification. The IIGSSA-DNN's REC and PRE are 0.92 outperformed by other algorithms. The IIGSSA-DNN is proven to be better at identifying positive samples. FPR is the rubric for judging percentage of correct positive samples. TPR is the accuracy rate for evaluating the correct classification of positive samples. FPR is a scale for judging the percentage of correct positive samples. PRE is an assessment of the accuracy of classifying positive samples correctly. The value of IIGSSA-DNN is 0.92 on all these rubrics, and the model is confirmed to have a strong performance in distinguishing between negative and positive samples. F1-M is a synthesis assessment index to assess the quality of the model. The F1-M value of IIGSSA-DNN is 0.92 higher than the other indexes. The model is confirmed to have higher model quality than the other models listed in this study. The AUC and *P*-values are represented in Figure 6. In Figure 6, the average ROC curve for each algorithm model is plotted as the blue curve. The AUC of IIGSSA-DNN has approached 1 and the *P* value is less than 0.05. The model has great statistical significance and better classification performance. Consequently, IIGSSA-DNN is exhibited to be a reliable classification model with much higher performance than the other models listed in this study.

## 5. Conclusion

DNN is a complex pattern recognition network system. The network structure and optimal weights have a drastic impact on the classification results of DNN. How to value the network structure and connection weights is a daunting task. In this study, a new chaotic map and Gaussian mutation are proposed to improve the optimal search strategy of SSA. The iterative chaotic map is validated by fourteen benchmark functions, and the iterative chaotic map improves SSA to a better rate than other chaotic maps. IIGSSA is identified as the best strategy to find the optimal fully connected weights of DNN. IIGSSA-DNN is contrasted with seven algorithms on the esophageal cancer dataset, and the seven algorithms are DNN, PSO-DNN, GSA-DNN, GWO-DNN, SSA-DNN, IIGSSA-KNN, and IIGSSA-SVM. The IIGSSA-DNN is proven to possess optimal performance in predicting the survival time of esophageal cancer patients. The accuracy of IIGSSA-DNN is substantially improved by the self-learning capability of DNN and the efficient search capability of IIGSSA. Therefore, IIGSSA-DNN gains more than traditional machine learning algorithms in dealing with complex issues. It is more accurate to solve the classification and recognition problems. According to this model, doctors can more keenly monitor the progress of each patient, thereby establishing a more benign diagnosis and treatment system. The parameters of the IIGSSA-DNN model rely on empirical selection, which limits the accuracy of prediction. The dataset sample samples used in this institute are relatively small, and the future period is verified in a large number of datasets and clinical trials. Taking the IIGSSA-DNN model as an opportunity, a secure smart device application is expected to be developed by researchers in the future. The application can bind the preliminary and retraining information of each patient, enabling doctors to customize personalized diagnosis and treatment schemes for each patient.

## Figures and Tables

**Table 1:** Fourteen benchmark functions.

Function name	Function	Dimension	Search space	Optimal value
Sphere	$F_{1}\left(x\right)=\overset{d}{\underset{i=1}{\sum }}x_{i}^{2}$	30	[−100, 100]	0
Schwefel 2.21	*F*_2_(*x*)=max{|*x*_*i*_|, 1 ≤ *i* ≤ *d*}	30	[−100, 100]	0
Schwefel 2.22	$F_{3}\left(x\right)=\overset{d}{\underset{i=1}{\sum }}\left|x_{i}\right|+\overset{d}{\underset{i=1}{\prod }}\left|x_{i}\right|$	30	[−10, 10]	0
Rosenbrock	$F_{4}\left(x\right)=\overset{d-1}{\underset{i=1}{\sum }}\left[100{\left(x_{i+1}-x_{i}^{2}\right)}^{2}+{\left(x_{i}-1\right)}^{2}\right]$	30	[−30, 30]	0
Step	$F_{5}\left(x\right)=\overset{d}{\underset{i=1}{\sum }}\left(\left[x_{i}+0.5\right]\right){}^{2}$	30	[−100, 100]	0
Quartic	$F_{6}\left(x\right)=\overset{d}{\underset{i=1}{\sum }}ix_{i}^{4}+random\left[0,1\right)$	30	[−1.28, 1.28]	0
Alpine	$F_{7}\left(x\right)=\overset{d}{\underset{i=1}{\sum }}\left|x_{i}\sin\left(x_{i}\right)+0.1x_{i}\right|$	30	[−10, 10]	0
Rastrigin	$F_{8}\left(x\right)=\overset{d}{\underset{i=1}{\sum }}\left(x_{i}^{2}-10\cos\left(2\pi x_{i}\right)+10\right)$	30	[−5.12, 5.12]	0
Sum squares	$F_{9}\left(x\right)=\overset{d}{\underset{i=1}{\sum }}ix_{i}^{2}$	30	[−10, 10]	0
Ackley	$F_{10}\left(x\right)=-20\exp\left(-0.2\sqrt{\frac{1}{d}\overset{d}{\underset{i=1}{\sum }}x_{i}^{2}}\right)-\exp\left(-0.2\sqrt{\frac{1}{d}\overset{d}{\underset{i=1}{\sum }}\cos\left(2\pi x_{i}\right)}\right)+20+e$	30	[−100, 100]	0
Maytas	*F*_11_(*x*)=0.26(*x*_1_^2^+*x*_2_^2^) − 0.48*x*_1_*x*_2_	2	[−10, 10]	0
Levi	*F*_12_(*x*)=sin^2^(3*πx*_1_)+(*x*_1_ − ^1^)(1+sin^2^(3*πx*_2_))+(*x*_2_ − ^1^)(1+sin^2^(2*πx*_2_))	2	[−10, 10]	0
Booth	*F*_13_(*x*)=(*x*_1_+2*x*_2_ − 7)^2^+(2*x*_1_+*x*_2_ − 5)^2^	2	[−10, 10]	0
Three-Hump	$F_{14}\left(x\right)=2x_{1}^{2}-1.05x_{1}^{4}+\frac{x_{1}^{6}}{6}+x_{1}x_{2}+x_{2}^{2}$	2	[−10, 10]	0

**Table 2:** Examine outcomes for fourteen benchmark functions.

Function	Algorithms	Optimal value	Mean value	Standard deviation
*F*_1_	SSA	**0**	8.037E-114	3.594E-113
	Tent	**0**	5.311E-202	**0**
	Chebyshev	**0**	7.017E-245	**0**
	Circle	**0**	**0**	**0**
	Iterative	**0**	4.101E-183	**0**
	Sine	**0**	1.298E-198	**0**
	Singer	**0**	7.994E-212	**0**
	Sinusoidal	**0**	1.202E-160	5.376E-160
	Logistic	**0**	4.07E-257	**0**
	Cubic	**0**	3.662E-163	2.223E-162

*F*_2_	SSA	**0**	3.856E-45	1.724E-44
	Tent	**0**	8.548E-155	3.823E-154
	Chebyshev	**0**	4.031E-130	1.803E-129
	Circle	**0**	1.481E-127	6.621E-127
	Iterative	**0**	**1.235E-156**	**4.303E-156**
	Sine	**0**	1.985E-136	8.877E-136
	Singer	**0**	9.621E-140	5.524E-140
	Sinusoidal	**0**	1.4722E-122	2.2693E-128
	Logistic	**0**	2.154E-133	1.312E-132
	Cubic	**0**	2.934E-133	2.0752E-79

*F*_3_	SSA	**0**	1.673E-27	7.483E-27
	Tent	**0**	2.714E-122	1.214E-121
	Chebyshev	**0**	9.729E-96	4.351E-95
	Circle	**0**	1.564E-74	6.994E-74
	Iterative	**0**	**5.909E-129**	**2.643E-129**
	Sine	**0**	2.99E-109	1.337E-108
	Singer	**0**	7.224E-105	3.231E-104
	Sinusoidal	**0**	1.316E-65	5.887E-65
	Logistic	**0**	1.106E-122	4.946E-122
	Cubic	**0**	1.165E-117	5.209E-117

*F*_4_	SSA	1.223E-05	1.142E-03	2.778E-03
	Tent	4.321E-08	5.348E-06	8.617E-06
	Chebyshev	5.946E-07	7.212E-05	8.992E-05
	Circle	1.033E-05	3.929E-04	5.017E-04
	Iterative	**2.255E-08**	**4.452E-06**	**6.736E-06**
	Sine	2.714E-07	1.764E-05	1.828E-05
	Singer	1.128E-06	7.727E-05	8.092E-05
	Sinusoidal	2.735E-04	1.373E-05	2.526E-04
	Logistic	1.344E-07	1.124E-05	1.206E-05
	Cubic	3.213E-08	1.333E-05	1.568E-05

*F*_5_	SSA	3486E-04	6.432E-03	5177E-03
	Tent	4.018E-07	9.14E-05	9.994E-05
	Chebyshev	1.981E-08	1.796E-05	1.97E-05
	Circle	4.387E-08	8.52E-04	9.895E-05
	Iterative	**3.0466E-08**	**1.774E-05**	**1.711E-05**
	Sine	1.496E-06	9.726E-04	9.667E-05
	Singer	1.618E-06	7.313E-04	8.238E-05
	Sinusoidal	3.918E-06	1.317E-04	1.371E-04
	Logistic	9.34E-06	7.424E-04	6.168E-04
	Cubic	7.205E-06	1.032E-04	1.236E-04

*F*_6_	SSA	3.1E-03	3.399E-02	2.059E-02
	Tent	2.912E-04	6.554E-03	4.036E-03
	Chebyshev	**7.183E-05**	4.367E-03	4.201E-03
	Circle	8.295E-04	8.557E-03	6.841E-03
	Iterative	3.63E-04	**4.008E-03**	**2.842E-03**
	Sine	1.218E-03	6.723E-03	4.694E-03
	Singer	5.581E-05	7.878E-03	5.441E-03
	Sinusoidal	5.971E-04	8.378E-03	7.758E-03
	Logistic	2.541E-04	7.907E-03	6.586E-03
	Cubic	4.965E-04	7.344E-03	4.835E-03

*F*_7_	SSA	**0**	5.837E-60	2.611E-59
	Tent	**0**	3.32E-119	1.485E-118
	Chebyshev	**0**	8.955E-90	4.005E-89
	Circle	**0**	1.03E-140	4.608E-140
	Iterative	**0**	9.454E-138	4.228E-137
	Sine	**0**	**2.375E-212**	**0**
	Singer	**0**	1.417E-102	6.335E-102
	Sinusoidal	**0**	3.156E-99	1.412E-98
	Logistic	**0**	5.296E-85	2.368E-84
	Cubic	**0**	3.802E-100	1.701E-99

*F*_8_	SSA	**1.008E-01**	2.237E-01	1.688E-01
	Tent	**1.008E-01**	1.011E-01	3.295E-02
	Chebyshev	**1.008E-01**	**1.009E-01**	**5.37E-05**
	Circle	**1.008E-01**	**1.009E-01**	1.034E-04
	Iterative	**1.008E-01**	**1.009E-01**	1.323E-04
	Sine	**1.008E-01**	**1.009E-01**	1.323E-04
	Singer	**1.008E-01**	**1.009E-01**	1.502E-04
	Sinusoidal	**1.008E-01**	**1.009E-01**	1.408E-04
	Logistic	**1.008E-01**	**1.009E-01**	1.608E-04
	Cubic	**1.008E-01**	**1.009E-01**	1.407E-04

*F*_9_	SSA	**0**	6.364E-105	3.486E-104
	Tent	**0**	5.818E-162	3.19E-161
	Chebyshev	**0**	2.456E-195	**0**
	Circle	**0**	1.067E-198	**0**
	Iterative	**0**	**2.373E-244**	**0**
	Sine	**0**	3.023E-112	1.656E-111
	Singer	**0**	2.521E-141	1.381E-140
	Sinusoidal	**0**	5.721E-140	3.133E-139
	Logistic	**0**	3.719E-203	**0**
	Cubic	**0**	3.758E-173	**0**

*F*_10_	SSA	**4.441E-16**	**4.441E-16**	**0**
	Tent	**4.441E-16**	**4.441E-16**	**0**
	Chebyshev	**4.441E-16**	**4.441E-16**	**0**
	Circle	**4.441E-16**	**4.441E-16**	**0**
	Iterative	**4.441E-16**	**4.441E-16**	**0**
	Sine	**4.441E-16**	**4.441E-16**	**0**
	Singer	**4.441E-16**	**4.441E-16**	**0**
	Sinusoidal	**4.441E-16**	**4.441E-16**	**0**
	Logistic	**4.441E-16**	**4.441E-16**	**0**
	Cubic	**4.441E-16**	**4.441E-16**	**0**

*F*_11_	SSA	**0**	4.594E-182	**0**
	Tent	**0**	1.434E-245	**0**
	Chebyshev	**0**	**0**	**0**
	Circle	**0**	5.773E-238	**0**
	Iterative	**0**	6.493E-226	**0**
	Sine	**0**	1.862E-243	**0**
	Singer	**0**	4.438E-315	**0**
	Sinusoidal	**0**	2.401E-145	1.074E-144
	Logistic	**0**	2.014E-282	**0**
	Cubic	**0**	1.378E-190	**0**

*F*_12_	SSA	2.324E-06	2.009E-05	9.768E-05
	Tent	1.093E-07	1.083E-05	1.845E-05
	Chebyshev	4.92E-07	5.578E-06	6.295E-06
	Circle	9.458E-08	6.071E-06	6.68E-06
	Iterative	1.19E-08	**3.015E-06**	**3.344E-06**
	Sine	**5.444E-09**	5.002E-06	4.757E-06
	Singer	2.386E-08	5.343E-06	5.608E-06
	Sinusoidal	4.846E-08	6.556E-06	5.957E-06
	Logistic	1.144E-07	6.205E-06	5.926E-06
	Cubic	2.044E-07	6.126E-06	8.436E-06
*F*_13_	SSA	2.647E-06	2.611E-05	2.979E-05
	Tent	9.904E-07	7.571E-04	8.334E-04
	Chebyshev	8.105E-07	1.441E-05	1.482E-05
	Circle	5.826E-07	1.133E-05	1.619E-05
	Iterative	5.674E-07	**1.009E-05**	**8.207E-06**
	Sine	**1.501E-08**	1.272E-05	1.293E-05
	Singer	1.144E-07	1.387E-05	1.481E-05
	Sinusoidal	2.314E-08	1.504E-05	2.021E-05
	Logistic	3.14E-08	1.363E-05	1.683E-05
	Cubic	2.296E-07	1.36E-05	1.427E-05

*F*_14_	SSA	**0**	4.752E-133	2.125E-132
	Tent	**0**	5.872E-268	**0**
	Chebyshev	**0**	7.727E-268	**0**
	Circle	**0**	2.862E-152	1.28E-151
	Iterative	**0**	**0**	**0**
	Sine	**0**	2.875E-311	**0**
	Singer	**0**	2.985E-122	1.335E-121
	Sinusoidal	**0**	1.003E-159	4.487E-159
	Logistic	**0**	9.108E-224	**0**
	Cubic	**0**	1.866E-260	**0**

**Table 3:** Continuous metrics in the dataset.

Variable	Mean	Median (range)	Variance
Tumor length	4.112	4 (1–11)	3.208
Tumor width	2.649	2.5 (0.3–9)	1.148
Tumor thickness	1.1776	1 (0.1–8)	0.471
WBC	6.5366	6.2 (2.5–13.6)	3.6958
LY	1.7622	1.8 (0–4)	0.3652
MONO	0.3899	0.4 (0–1.4)	0.06661
NEUT	4.0011	3.7 (0–9.8)	2.8097
EOS	0.1238	0.1 (0–0.9)	0.0198
BASO	0.04163	0 (0–5)	0.005549
RBC	4.43	4.48 (2.73–5.75)	0.2289
HB	137.4347	138 (64–169)	223.7577
PLT	236.8518	231 (100–448)	52.606
TP	71.0377	71 (50–92)	54.4092
ALB	42.0201	42 (26–59)	25.1281
GLB	29.1533	29 (16–45)	28.8656
PT	10.2271	10.2 (7–16.6)	2.4610
APTT	35.9095	35.1 (15.4–62.2)	52.9934
TT	15.3420	15.5 (10.9–21.3)	2.9607
FIB	387.3433	378.3960 (167.613–774.433)	985.7021
Age	60	60 (38–82)	70.099
Survival time	4	3 (0–11)	12.873

The unit of tumor length, tumor width, tumor thickness is centimeter. The unit of WBC, LY, MONO, NEUT, EOS, BASO, RBC, and PLT is 109/L. The unit of HB, TP, ALB, and GLB is g/L. The unit of PT, APTT, and TT is second(s). The unit of FIB is mg/dL. The unit of survival time is year.

**Table 4:** Discrete metrics in the dataset.

Project	Category	Number of population	Percentage of population (%)
Gender	Male	247	62
	Female	151	38

Degree of differentiation	Poorly differentiated	158	40
	Moderately differentiated	217	54
	Highly differentiated	23	6

Tumor site	Lower thoracic	78	20
	Mid thoracic	267	67
	Upper thoracic	53	13

Transfer situation	Negative	200	50
	Positive	198	50

TNM stages	I	39	10
	II	172	43
	III	166	42
	IV	21	5

Survival status	Live	101	25
	Dead	297	75

**Table 5:** Results of the predictive model evaluation.

Algorithms	Acc	FPR	REC	PRE	TNR	F1-M	AUC	*P*
DNN	0.76	0.16	0.76	0.76	0.76	0.76	0.76	0.03
PSO-DNN	0.79	0.133	0.79	0.79	0.79	0.79	0.79	0.04
GSA-DNN	0.83	0.133	0.83	0.83	0.83	0.83	0.83	0.04
GWO-DNN	0.81	0.117	0.81	0.81	0.81	0.81	0.81	0.01
WOA-DNN	0.86	0.140	0.86	0.86	0.86	0.86	0.86	0.01
SSA-DNN	0.89	0.147	0.89	0.89	0.89	0.89	0.89	0.04
IIGSSA-KNN	0.75	0.21	0.75	0.75	0.75	0.75	0.75	0.04
IIGSSA-SVM	0.82	0.18	0.82	0.82	0.82	0.82	0.82	0.04
IIGSSA-DNN	0.92	0.100	0.92	0.92	0.92	0.92	0.92	0.01

## Data Availability

The datasets presented in this article are not readily available because the data used in the study are private and confidential data. The datasets' access can be obtained from the corresponding author upon request.
